# Parametric decay induced first-order phase transition in two-dimensional Yukawa crystals

**DOI:** 10.1038/s41598-022-24988-8

**Published:** 2022-11-28

**Authors:** Srimanta Maity, Garima Arora

**Affiliations:** 1 Department of Physics, Indian Institute of Technology Delhi, Hauz Khas, New Delhi, 110016 India; 2 Institute for Plasma Research, Bhat, Gandhinagar, 382428 India

**Keywords:** Plasma physics, Phase transitions and critical phenomena

## Abstract

The melting process of two-dimensional (2D) Yukawa crystals for dusty plasma medium induced by external perturbations has been explored using molecular dynamics simulations. A 2D monolayer of particles interacting via Yukawa pair potential is formed in the presence of an external confinement potential. The confinement potential is a combined effect of the gravitational force and an externally applied electric force, which mimics the sheath electric field in dusty plasma experiments. The response of the 2D crystalline layer to an external perturbation is investigated. It is shown that transverse surface waves are generated below a particular threshold value of initial perturbation, but the crystalline order remains. However, above a threshold value of initial disturbance, the crystalline order structure of the 2D layer breaks, and it melts. The melting process is shown to be a first-order phase transition. We have demonstrated that the nonlinear amplitude modulation of initial disturbance through the parametric decay instability is responsible for the melting. Our proposed mechanism of first-order phase transition in the context of 2D dusty plasma crystal is distinctly different from the existing theoretical models. This research can provide a deeper understanding of the experimental observations in the context of plasma crystal.

## Introduction

Phase transition in a two-dimensional system has remained an unresolved mystery and an interesting research topic^1–3^. Two-dimensional systems can be realized in different contexts of physics, e.g., electrons on the surface of liquid helium^4,5^, ionic crystal^6^, colloidal medium^7,8^, and dusty plasma systems^9–11^. Melting in two-dimensional (2D) crystal follows a different mechanism from three-dimensional (3D) crystal and is a source of disputation. Mainly two theories have come up in the past that tried to interpret the experimental findings. The first is Kosterlitz–Touless–Halperin–Nelson–Young (KTHNY) theory^1^ which predicts a two-step melting process where the 2D crystal first transforms to an intermediate hexatic phase and then finally comes into a liquid phase. The phase transition is caused by the dislocation and disclination leading to the breaking of long-range order called the hexatic phase. The intermediate phase formation is continuous and leads to the second-order phase transition. Another well-known theory is grain-boundary induced (GBI)^2^ melting, which predicts a first-order phase transition without the appearance of a hexatic phase.

Dusty plasma medium is an ideal model system for studying the phase transition in 2D. This is because the time and length scales associated with the response of a dusty plasma medium are of the order of human perceived scales and can be easily diagnosed in laboratory experiments. Dusty plasma medium consists of micron or sub-micron sized negatively charged particles in a plasma environment. These micron-sized charged particles can form a two-dimensional (2D) and three-dimensional (3D) ordered structure (also known as plasma crystal) under certain conditions, which are easily achievable in experiments. There are several experimental as well as theoretical studies on the formation of dusty plasma crystal^12–15^, static and dynamical phase behaviour of crystalline structure^16–18^, crystal cracking induced by energetic particles^19^, cluster formation^20–22^, and various dust lattice modes^23–25^.

Various experiments and simulations have been performed to study the characteristics, order, and origin of phase transition in 2D plasma crystals. Nosenko et al.^26^ experimentally observed the melting of laser-heated dust crystals which follows GBI theory. Recently, Vasilieva et al.^27^ used a laser to study the melting process induced in a 2D plasma crystal and showed clear-cut evidence of two-stage melting following KTHNY theory. Sheridan^28^ performed numerical simulations of the experimental observations of melting of a 2D plasma crystal and the results agreed well with the experimental results and the KTHNY theory. Melzer et al.^9^ experimentally observed the melting of a two-layer plasma crystal and reported that their experimental findings could not be explained either by KTHNY^1^ theory or GBI^2^ theory of melting. A host of other works have also been reported and have shown that plasma-induced instability could be the cause of the melting of a 2D dust crystal. Joyce et al.^29^ showed the first-order phase transition and demonstrated that ion dust two-stream instability is responsible for the melting of 2D crystal. Schwiggert et al.^30^ observed the self-excited vertical oscillation induced by plasma instability causes the melting of crystals. Samsonav et al.^10^ showed the melting of a 2D plasma crystal from a shock wave. Another well-known mechanism of instability-triggered phase transition is Mode Coupling Instability (MCI) originating from the ion wakes. The MCI occurs when the flowing plasma forms the ion wakes and interacts with the dust particles through the nonreciprocal interaction. Thus, the system becomes non-Hamiltonian, and energy from the flowing ions is converted into the kinetic energy of micro-particles initiating the melting of the entire crystal. Ivlev and Morfill^25^ provided a theory of MCI for the first time where they showed the resonant coupling of Dust-Lattice (DL) modes in the presence of ion wakes could trigger an instability. Couëdel et al.^31^ reported the first ever experimental observation of melting of a 2D plasma crystal due to mode coupling. However, Liu et al.^32^ experimentally demonstrated that the coupling of dust lattice modes could occur in a perfectly stable dusty plasma crystal, without melting. In a nutshell, the universality in the melting behavior of a 2D complex plasma crystal is still uncertain.

In the present work, we have suggested a new mechanism of first-order phase transition which is distinctly different from the past theoretical models. We have explored the first-order phase transition of a 2D plasma crystal confined in a parabolic potential well using 3D MD simulations. In particular, we have studied the response of a 2D crystalline monolayer to an externally imposed initial perturbation. The initial disturbance is induced by displacing some particles at the center in the downward direction. The perturbed particles radiate their energy by circular transverse waves propagating outward in the x–y plane. However, the crystalline structure melts with a first-order phase transition above a threshold value of initial perturbation. The structural properties have been characterized using the Voronoi diagram and the pair correlation function. The sharp jump or discontinuity in various parameters like the Lindemann ratio, order parameter, and Coulomb coupling parameter confirms that the order of phase transition is first-order. Thus, the KTHNY theory of melting associated with the intermediate hexatic phase (second-order phase transition) could not explain our simulation observations. We have observed that the meting is initiated at the center of the monolayer and propagates radially outward. Hence, the first-order melting transition observed in our study is dissimilar from the characteristics of the GBI theory. Also, in our case, the absence of anisotropic ion-wake potential rules out the possibility of MCI^25^. We have demonstrated using MD simulations that the parametric decay instability is responsible for the melting of a crystalline plane. Parametric Decay Instability (PDI) of dust lattice waves has been studied theoretically by Shukla^33^ showing a phase transition of a dusty plasma medium from solid to gas-like state. PDI are also observed in many other aspects of plasma physics, e.g., ionosphere^34^, inertial confinement fusion^35^, magnetic confinement fusion^36^, laser-plasma interactions^37,38^, laser wake field acceleration^39^. The PDI is the nonlinear process of transferring the energy of a pump wave into other waves. Any mode can participate in PDI if it crosses a certain threshold of nonlinearity. Parametric decay instability is closely related to the modulational interactions^40^. In our simulations, we have shown that the amplitude of initial perturbation gets modulated through PDI, and above a threshold value of perturbation, it initiates melting.

This paper has been organized as follows. First, we describe the simulation setup. Then, in various subsections the response of a 2D plasma crystal to an external perturbation has been discussed. Initially, we show the generation of transverse circular waves and a first-order phase transition from solid to liquid phase. Later, we describe the origin of phase transition. Finally, we provide a summary of this work.

## MD simulation details

**Figure 1:**
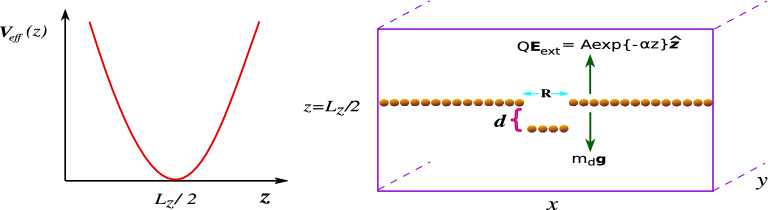
The schematic of the simulation setup has been shown here. Forces due to gravity ($m_dg$) and due to the externally applied electric field ($QE_{ext}$) have been applied in the vertically opposite directions (i.e., $\pm {{\hat{z}}}$), resulting in the formation of a monolayer crystal. The effective external potential energy $V_{eff}$ contributed from gravity, and externally applied electric potential has a parabolic shape along ${{\hat{z}}}$ with a minimum located at $z = L_z/2$. Boundary conditions in the *x*–*y* plane are considered to be periodic. Particles within a small circular region of radius *R* in the central portion of the monolayer crystal are displaced vertically by a distant *d* from their equilibrium positions.

In this work, three-dimensional (3D) molecular dynamics (MD) simulations have been carried out to investigate the response of a 2D crystal under external perturbations. An open-source classical MD code LAMMPS^41^ has been used for this purpose. Initially, ten thousands identical point particles, representing dust grains, are randomly distributed in a 3D simulation box with lengths $L_x = L_y = L_z = 10$ cm in ${{\hat{x}}}$, ${{\hat{y}}}$, and ${{\hat{z}}}$ directions, respectively. The system parameters considered in our simulation study are as follows^42^. The charge and mass of the particles are chosen to be $Q = 10000e$, and $m_d = 5\times 10^{-13}$ Kg, respectively. Here, *e* is the charge of an electron. The plasma Debye length is considered to be $\lambda _D = 1.128 \times 10^{-3}$ m. We have also carried out simulations with different values of $\lambda _D$ and found that the physical phenomena presented here remains the same. For our chosen values of number of particles ($N = 10000$) and lengths of the simulation box in *x*–*y* plane (i.e., $L_x$ and $L_y$), the 2D number density of the monolayer is given by $n = 1.0 \times 10^6$ $\hbox {m}^{-2}$, which corresponds to an average inter-particle distance $a = 1/\sqrt{n\pi } = 5.64 \times 10^{-4}$ m. Thus, the value of the screening parameter is calculated to be $\kappa = a/\lambda _D = 0.5$. For these parameters, the characteristics dust plasma frequency is given by $\omega _{pd} = 22.668$ Hz. The time steps of the simulation runs are considered as $dt = 0.01\omega _{pd}^{-1}$, which is small enough to resolve the fastest dynamics associated with the dust grains. In our simulations, particles interact via Yukawa or screened Coulomb pair potential, $U(r) = (Q/4\pi \varepsilon _0r)\exp {(-r/\lambda _D)}$. Here, $\varepsilon _0$ represents the electric permittivity in free space. In addition to the Yukawa pair interaction, particles are also subjected to the force due to gravity, $m_d{g}$ ($\hat{-z}$) acting vertically downward, and the force associated with an externally applied electric field $Q{\mathbf {E}}_{ext} = A\exp {-\alpha z}$ (${{\hat{z}}}$) acting vertically upward for the negatively charged particles^15^. These two forces provide a vertical confinement potential with a parabolic form, as shown in Fig. 1. We have chosen the parameters *A* and $\alpha$ so that particles organize themselves in a 2D monolayer in the *x*–*y* plane levitating at a height $z = L_z/2$^15^. The boundary conditions in ${{\hat{x}}}$ and ${{\hat{y}}}$ directions are taken to be periodic.

In our simulations, we have used a Nose Hoover thermostat^43,44^ to thermally equilibrate the system with the desired temperature $T_0$ associated with the Coulomb coupling parameter $\Gamma = Q^2/4\pi \varepsilon _0k_BT_0 = 1000$. Here, $k_B$ is the Boltzmann constant. Once the system reaches the thermal equilibrium state, we have disconnected the thermostat and let the system evolve in a micro-canonical (NVE) ensemble where the total number of particles *N*, the volume of the simulation box *V*, and total energy *E* remain constant. The time scales, length scales, and energies are normalized by $\omega _{pd}^{-1}$, *a*, and $k_BT_0$, respectively.

## Results and discussion

Initially, we obtained a hexagonal monolayer crystalline structure using MD simulation. For our chosen values of parameters, e.g., $\kappa$ and $\alpha$, this monolayer levitates at the location of minimum (i.e., $z = L/2$) of the effective external potential energy $V_{eff}$. The $V_{eff}$ has contributions from gravitational energy and energy associated with the externally applied electric field in the vertical (${{\hat{z}}}$) direction. The profile of $V_{eff}$ as a function of *z* has been shown by the schematic in Fig. 1, and it is seen that $V_{eff}$ has a parabolic shape. We have perturbed a few particles initially located within a small radius *R* from the center of the monolayer structure by displacing them with a distance *d* along $-{{\hat{z}}}$ direction. The schematic has clearly illustrated this in Fig. 1. These perturbed particles will start a vertical oscillation around the monolayer under effective parabolic potential $V_{eff}$. Their motion will induce a disturbance in the monolayer crystalline structure. We have presented various features of our observations in the following subsections.

### Surface wave generation and first-order phase transition

**Figure 2:**
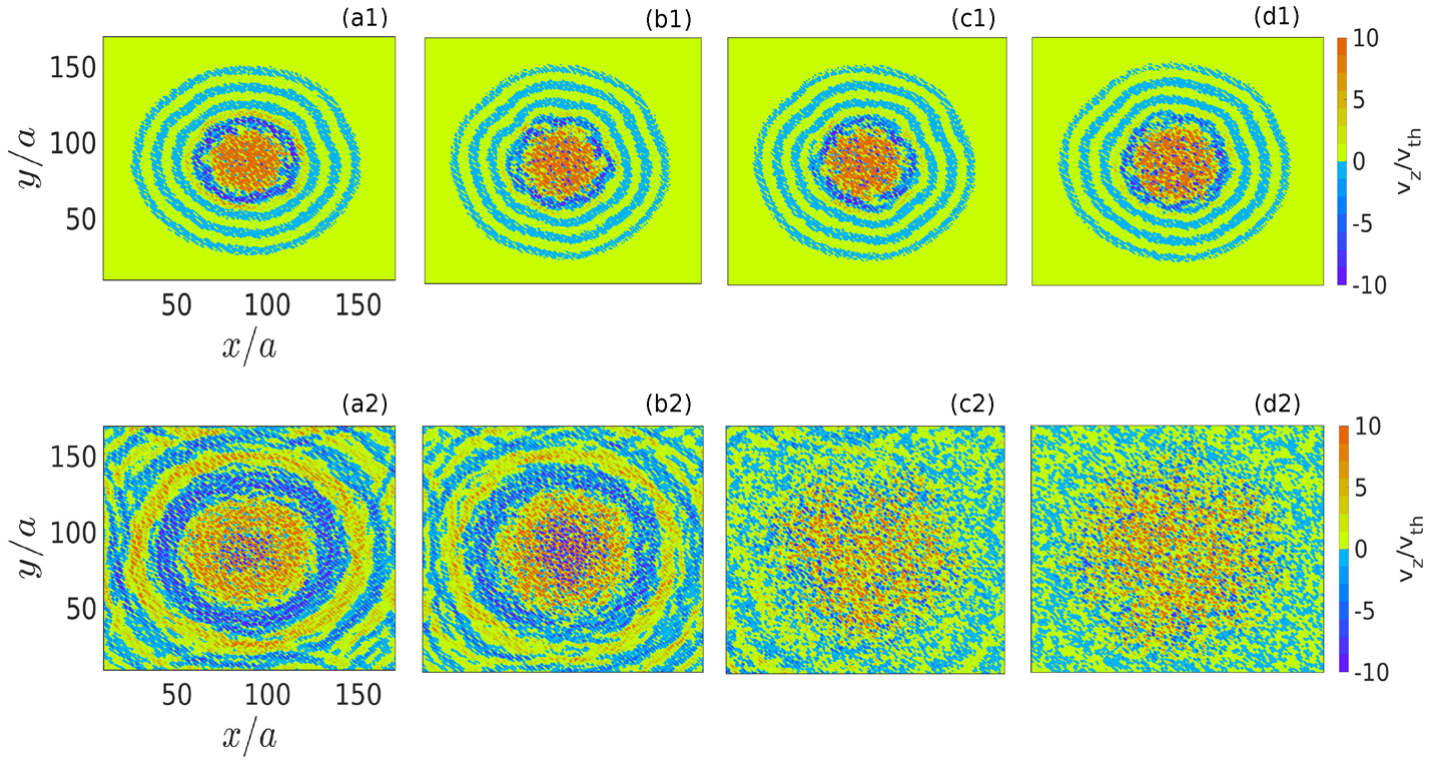
The profile of the vertical component of velocity $v_z$ in the *x*–*y* plane has been shown here by the pseudo-color plot. In the subplots (**a1**)–(**d1**), we have shown $v_z$ profiles for four different simulation runs with (**a1**) $d/a = 1.22$, (**b1**) $d/a = 1.50$, (**c1**) $d/a = 1.51$, and (**d1**) $d/a = 1.72$ at a particular time $\omega _{pd}t = 200$. The same at a particular simulation time $\omega _{pd}t = 1000$ has been shown in subplots (**a2**)–(**d2**), respectively. Here, the velocities are normalized by equilibrium thermal velocity ($v_{th}=\sqrt{k_BT_0/m_d}$) of dust particles.

The particles displaced from the equilibrium monolayer crystal acquire oscillating vertical velocity due to the restoring force of the parabolic potential well. The amplitude of their velocity increases with an increase of initial displacement *d*. However, the frequency of their vertical oscillations only depends upon the profile of $V_{eff}$. The parameter $\alpha$ defines the sharpness of $V_{eff}$. Thus, for a particular value of *Q* and $m_d$, as long as $\alpha$ is kept constant, vertical oscillation frequency does not change with the value of initial displacement *d*. As the initially displaced particles in the central regime of the monolayer start to oscillate around it, they impart their energy to the surrounding particles residing in the monolayer crystal via pair interactions. As a result, a transverse surface wave is initiated, spreading in the x–y plane of the monolayer from its central regime. This has been demonstrated in Fig. 2. The space distributions of $v_z$ in the *x*–*y* plane have been shown in Fig. 2 at two particular instants of time $\omega _{pd}t = 200$ and 1000 for four different simulation runs with changing values of *d*. It is seen from the pseudo-color plots in subplots (a1)–(d1) of Fig. 2 that at time $\omega _{pd}t = 200$ for all the four cases, the ${{\hat{z}}}$ component of particle’s velocity $v_z$ forms circular wavefronts in the x–y plane. This is because transverse surface waves with particle’s motions in the vertical directions ($\pm {{\hat{z}}}$) have been initiated and spread up to a certain radius beyond the initially perturbed circular region in the plane of monolayer crystal. The later stage of the evolution at time $\omega _{pd}t = 1000$ has been shown in subplots (a2)–(d2) of Fig. 2. It has been observed that for $d = 1.22a$ and $d = 1.50a$, circular wavefronts are continue forming and spreading away from the perturbed region, as shown in subplots (a2) and (b2) of Fig. 2. Whereas for $d = 1.51a$ and $d = 1.72a$, we have observed that the regular circular fronts of $v_z$ are not there. Instead, $v_z$ is distributed randomly in the *x*–*y* plane, as illustrated in subplots (c2) and (d2). It is also seen from these two pseudo-color plots that the particles at the central regime remain more energetic than the outer portion of the monolayer crystal. The supplementary videos *(.mpg files)* created using VMD^45^ illustrate particle trajectory evolutions for $d/a = 1.50$ and $d/a = 1.51$, respectively.

**Figure 3:**
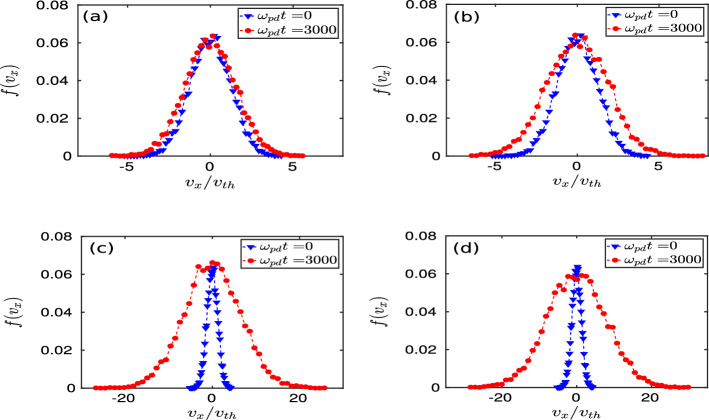
Velocity distribution functions $f(v_x)$ for the *x*-component of velocity $v_x$ have been shown at two different instants of time $\omega _{pd}t = 0$ (blue triangles), and $\omega _{pd}t = 3000$ (red dots). In subplots (**a**)–(**d**), the $f(v_x)$ have been evaluated for the simulation runs with $d/a = 1.22$, 1.50, 1.51, and 1.72, respectively.

To further analyze, we have also probed the ${{\hat{x}}}$ and ${{\hat{y}}}$ components of velocity of the particles forming monolayer crystal. The velocity distribution functions $f(v_x)$ associated with ${{\hat{x}}}$-component of velocity ($v_x$) have been evaluated for different simulation runs with changing values of *d* and are shown in Fig. 3. The distribution functions associated with the ${{\hat{y}}}$-component of velocity are the same as $f(v_x)$ because of the symmetry in the x–y plane. We have evaluated the $f(v_x)$ for two different instants of time in each cases, as shown in Fig. 3 by the blue-marked line for $\omega _{pd}t = 0$ (initial time of perturbation) and red-marked line for $\omega _{pd}t = 3000$ (final steady-state). The distribution functions have remained unchanged except for a slight broadening from their initial profiles for $d = 1.22a$ and 1.50*a*, as illustrated in subplots (a) and (b), respectively. However, it is interesting to see from subplots (c) and (d) that $f(v_x)$ has broadened significantly in simulation runs with $d = 1.51a$ and 1.72*a*. Thus, the root-mean-square (RMS) values of $v_x$ have increased in time for $d = 1.51a$ and 1.72*a*, and the same is true for $v_y$ too, indicating an increase in temperature of the monolayer.

**Figure 4:**
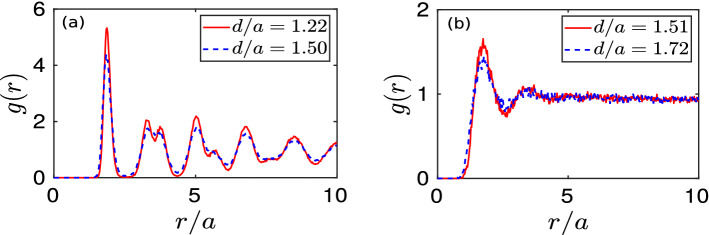
Pair correlation functions (*g*(*r*)) evaluated at a particular time $\omega _{pd}t = 3000$ have been shown in subplots (**a**) and (**b**) for four different simulation runs with $d/a = 1.22$, 1.50, 1.51, and 1.72.

It would be interesting to see the effect of the initial perturbation on the structural properties of the monolayer crystal. For this purpose, we have evaluated pair correlation function *g*(*r*) at a particular time $\omega _{pd}t = 3000$ for different simulation runs with changing values of initial perturbation i.e., *d*. The pair correlation function, also known as the radial distribution function, is an important parameter identifying the long-range spatial order in an arrangement of particles. It is defined as,

1 $$\begin{aligned} g(r) = \bigg \langle \frac{N_r(r, dr)}{\rho 2\pi r dr} \bigg \rangle , \end{aligned}$$

where $\langle ... \rangle$ represents the ensemble average. Here, $N_r (r, dr)$ defines the number of particles can be found within a circular strip between radius *r* and $r + dr$ away from a reference particle. The parameter $\rho$ represents the average number density of the monolayer, i.e., $N/(L_xL_y)$, where *N* is the total number of particles forming the monolayer. Sharp multiple periodic peaks in the profile of *g*(*r*) in each cases for $d = 1.22a$ and 1.50*a* is observed, as illustrated in subplot (a) of Fig. 4. This confirms that in these cases, the initial crystalline order structures are still retained at $\omega _{pd}t = 3000$ after the initial perturbation. Whereas for the simulation runs with $d = 1.51a$ and 1.72*a*, it is seen that only the first two peaks appear in the profile of *g*(*r*) demonstrating a typical characteristic of a liquid, as depicted in subplot (b) of Fig. 4. We have also estimated the bond order parameter $\psi _6$ by calculating the local bond angle for each particle with its neighboring particles and then averaged over all the particles. At $\omega _{pd}t = 3000$, for $d/a = 1.22$ and 1.50, its value comes out to be 0.88, representing hexagonal crystalline structure. Whereas, for simulation runs with $d/a = 1.51$ and 1.72, the value of $\psi _6$ comes out to be 0.1, typically defines a liquid state.

**Figure 5:**
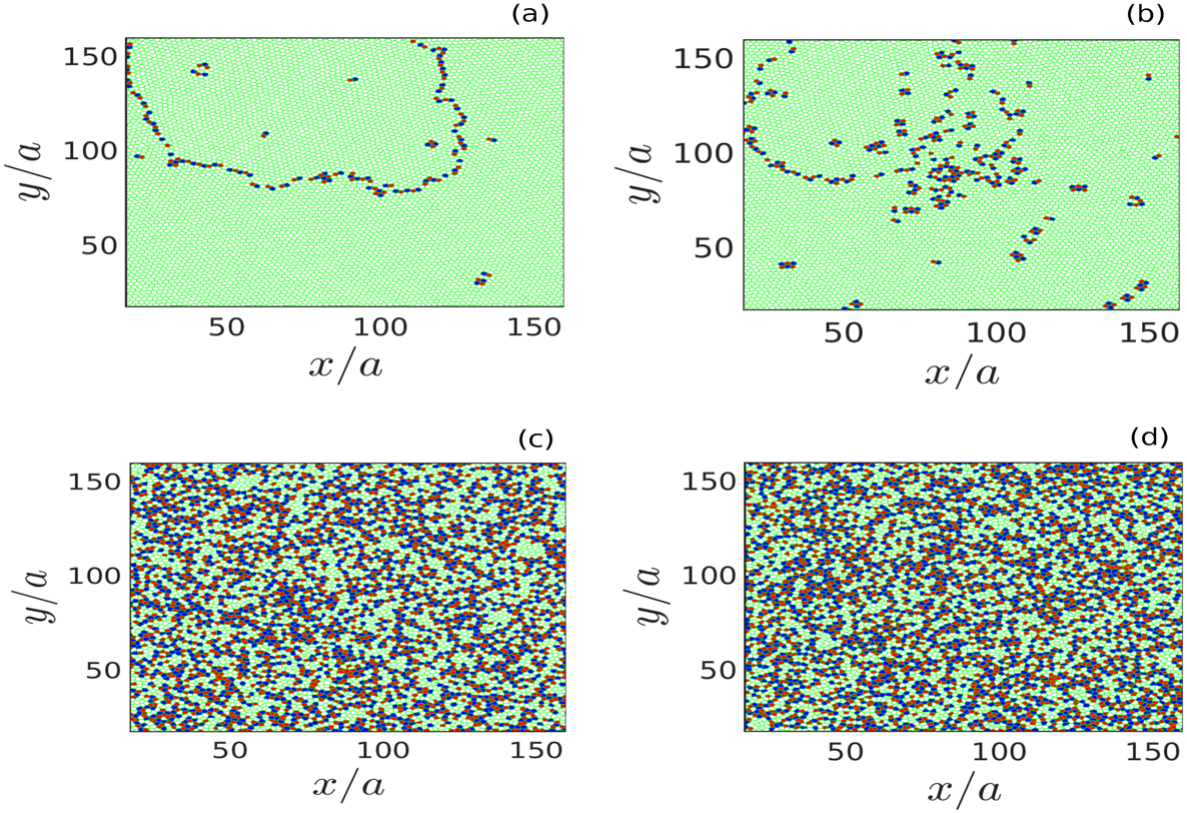
Voronoi diagrams obtained from the particle configuration in the x–y plane have been shown for the cases with (**a**) $d/a = 1.22$, (**b**) $d/a = 1.50$, (**c**) $d/a = 1.51$, and (**d**) $d/a = 1.72$ at a particular time $\omega _{pd}t = 3000$. Here, the green color patches represent the hexagonal symmetry. Color patches except green have represented Voronoi cells which are not hexagonal.

To further analyze the effect on the structural configuration of particles under the influence of initial perturbation, we have constructed Voronoi diagrams using (*x*, *y*)-coordinates of particles obtained in different simulation runs with changing values of *d*. A comparison of the nature of Voronoi diagrams for four different values of *d* can be seen in subplots (a)–(d) of Fig. 5. The hexagonal Voronoi cells are marked by green color. The red, blue, and cyan color patches correspond to other polygons. It is seen that for $d = 1.22a$ and 1.50*a*, hexagonal structures dominate throughout the Voronoi diagrams, as demonstrated in subplots (a) and (b) of Fig. 5. However, for the simulation runs with $d = 1.51a$ and 1.72*a*, the hexagonal symmetry is destroyed. The five-fold, seven-fold, and other Voronoi cells appear throughout the Voronoi diagrams, as can be seen in subplots (c) and (d) of Fig. 5. These observations confirm the findings in the pair correlation function analysis shown in Fig. 4. The outcomes of structural analysis using the pair correlation function and Voronoi diagrams demonstrate that above a critical value of *d* (e.g., $d>1.50a$), both short-range and long-range orders of particle’s arrangement in the monolayer crystal are destroyed, indicating a crystalline to a liquid phase transition.

**Figure 6:**
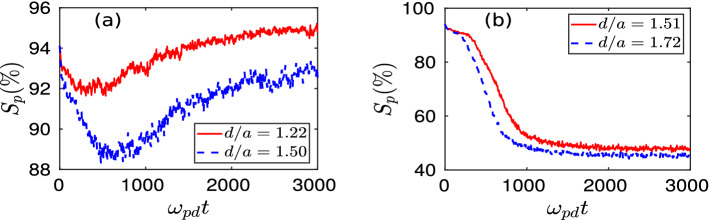
Time evolutions of structural order parameter $S_p$ (in $\%$) for four different values of *d* have been shown in subplots (**a**) and (**b**).

To further explore this phase transition process, we have calculated a structural order parameter ($S_p$), defined as $S_p = (N_{hc}/N_{tc}) \times 100$ (in $\%$), from the Voronoi diagram analysis. Here, $N_{hc}$ is the number of hexagonal cells, and $N_{tc}$ defines the total number of polygons in the Voronoi diagram. The time evolution of $S_p$ calculated in different simulation runs is shown in Fig. 6. It is seen that for $d = 1.22a$ and $d = 1.50a$, order parameter $S_p$ decreases initially with time and attains a minimum value. As time evolves, it is observed that $S_p$ starts to increase from its minimum value in each case, with the final values remaining above $90 \%$. This has been illustrated in subplot (a) of Fig. 6. Whereas, for the simulation runs with $d = 1.51a$ and $d = 1.72a$, it is observed that as time evolves there is a steady decrease in $S_p$ from its initial value ($\approx 95 \%$) and finally saturates at a very low value ($\approx 45 \%$), as depicted in subplot (b) of Fig. 6. These results are consistent with the outcomes of structural analysis using *g*(*r*) and the Voronoi diagram. This is another demonstration of crystal to liquid phase transition initiated due to the initial perturbation above a critical value of *d*.

**Figure 7:**
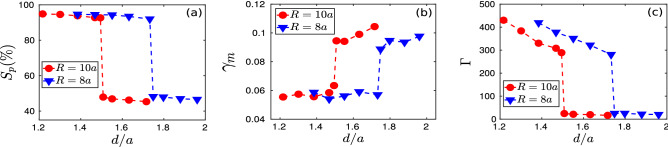
Variations of (**a**) structural order parameter $S_p$ (in $\%$), (**b**) Lindemann parameter $\gamma _{m}$, and (**c**) coupling parameter $\Gamma$ with displacement *d* have been shown for two different radii of the perturbed region, $R = 10a$, and 8*a*.

To ascertain the order of the phase transition, a large set of simulations were carried out with varying vertical displacement (*d*) of the particles initially located at the central regime of the monolayer crystal. In these simulation runs, we had chosen two cases of initial perturbations to support our observations by considering the radius of the perturbed region to be $R = 8a$ and 10*a*. We have evaluated three different parameters: (i) structural order parameter $S_p$, (ii) Lindemann parameter $\gamma _m$, and (iii) Coulomb coupling parameter $\Gamma$ to characterize the nature of the phase transition. All these three parameters have been calculated at the final steady-state of the simulation runs and are averaged over time. The structural order parameter ($S_p$) representing the fraction of the hexagonal structures present in the particle configuration has been shown in the subplot (a) of Fig. 7. It is seen that there is a sudden jump in the order parameter $S_p$ after a certain threshold value ($d_{th}$) of *d*. This happens in each cases, i.e., both $R = 8a$ and 10*a*. This clearly demonstrates a first-order phase transition resulting in the melting of the monolayer crystal. However, the value of $d_{th}$ decreases with an increase of radius *R*. Another first-hand diagnostic tool to identify the melting of a crystal is the Lindemann parameter representing the root-mean-square amplitude of thermal vibration^46,47^. It is defined as^48^,

2 $$\begin{aligned} \gamma _m = \frac{\sqrt{<(dr-<dr>)^2>}}{\Delta }, \end{aligned}$$

where *dr* represents the distance between two neighboring particles and $\Delta$ defines the average lattice constant obtained from the position of the first peak of *g*(*r*) in the corresponding crystalline phase. In the subplot (b) of Fig. 7, we have shown the variation of Lindemann parameter $\gamma _m$ with the changing values of *d*. In this case, it is seen that after a threshold value of *d*, $\gamma _m$ increases abruptly due to a slight increase of initial perturbation *d*. This phenomenon also happens for the coupling parameter $\Gamma$, as shown in the subplot (c) of Fig. 7. In these cases also, it is seen that $d_{th}$, the threshold value of *d* at (or above) which the first-order melting transition takes place, decreases with the increase of *R*. Thus, in conclusion, a first-order melting transition occurs in a monolayer Yukawa crystal above a certain threshold value of initial perturbation. This threshold value decreases with the strength of the perturbation. The fundamental origin behind this phase transition has been discussed in the following subsection.

### Amplitude modulation via parametric decay

To identify the origin of melting at the particle level, we randomly chose a particle initially located within the perturbed circular region of radius $R=10a$. We have tracked the *z*-coordinate of this particle with time for two different simulation runs with $d/a = 1.50$ and $d/a = 1.51$. It is to be noticed that the effective external potential energy has a parabolic form along ${\hat{z}}$, as has been shown in Fig. 1. Thus, if we displaced a particle vertically, it should oscillate sinusoidally along ${\hat{z}}$ with a particular frequency depending upon the parameter $\alpha$ representing the strength of the vertical confinement potential.

**Figure 8:**
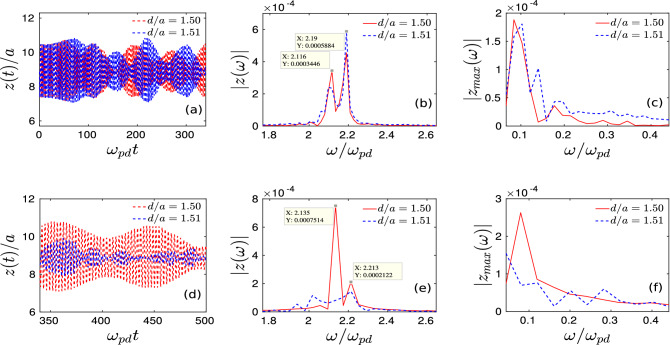
The time evolution of *z*-coordinate and the corresponding Fourier spectra of a randomly chosen particle, which was initially located within the radius $R = 10a$, have been shown for two different initial perturbations with $d/a = 1.50$, and $d/a = 1.51$. We have considered two cases, Case-I and Case-II. In Case-I, we have considered the time from $\omega _{pd}t = 0-340$. The time series data of *z*(*t*) (**a**), the absolute of Fourier spectrum of *z*(*t*) (**b**), and $z_{max}(t)$ (**c**). Subplots (**d**)–(**f**) represent the same for Case-II, where we have considered the time to be in between $\omega _{pd}t = 340-500$.

**Figure 9:**
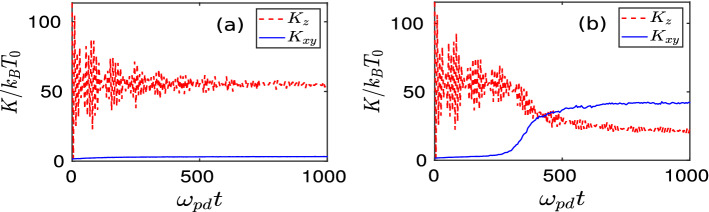
Time evolution of kinetic energy $K_z$, associated with the ${{\hat{z}}}$-component of the velocity (red dashed line) and $K_{xy}$, associated with the ${{\hat{x}}}$ and ${{\hat{y}}}$-components of the particle’s velocity (blue solid line) have been shown for (**a**) $d/a = 1.50$ and (**b**) $d/a = 1.51$. The kinetic energies are normalized by $k_BT_0$ where $k_B$ is the Boltzmann constant and $T_0$ is the equilibrium temperature of dust particles.

**Figure 10:**
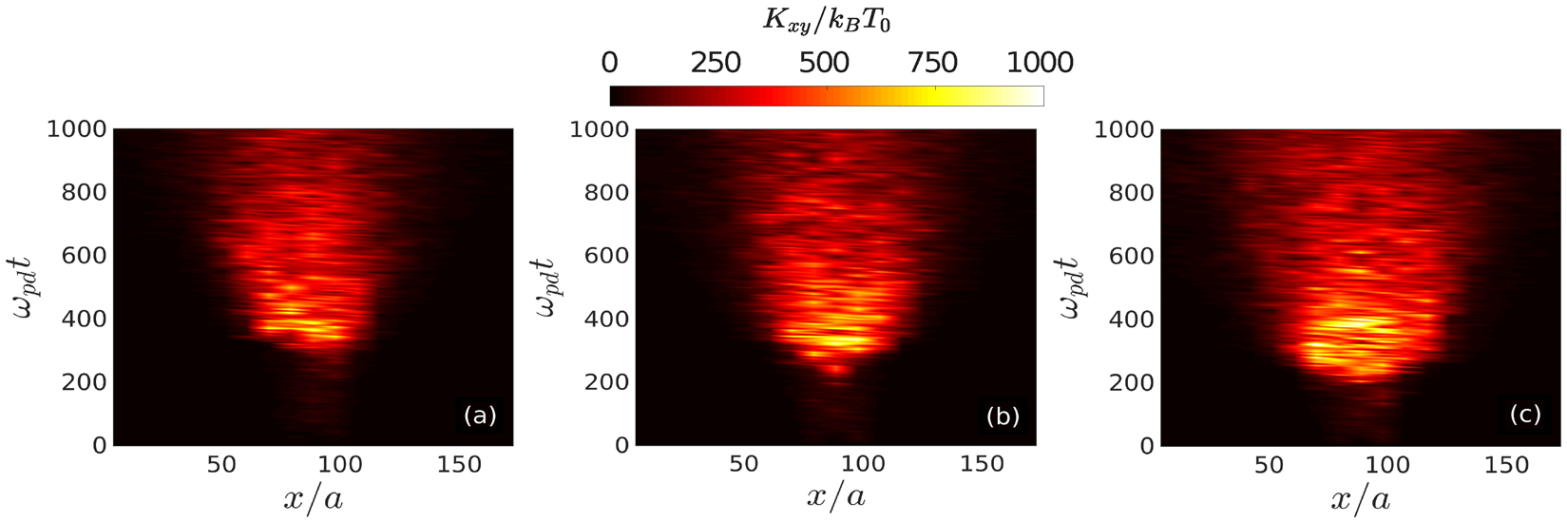
The distributions of in-plane kinetic energy $K_{xy}$ in *x*–*t* plane, averaged over a narrow strip along ${{\hat{y}}}$ around the middle of the *x*–*y* plane, have been shown for (**a**) $d/a = 1.51$, (**b**) $d/a = 1.63$, and (**c**) $d/a = 1.72$.

The time evolution of the *z*-coordinate of the chosen particle and the corresponding Fourier spectra have been depicted in Fig. 8 for two cases, (i) Case-I and (ii) Case-II. In Case-I, we have tracked the time history of *z*-coordinate at the initial stage of evolution, i.e., from time $\omega _{pd}t = 0$ (time of the initial perturbation) to $\omega _{pd}t = 340$. Case-II represents the dynamics at the later stage of the simulation run, i.e., from time $\omega _{pd}t = 340$ to $\omega _{pd}t = 500$. In both cases, we have evaluated the Fourier spectra of *z* and $z_{max}$, i.e., maximum values of *z* from the corresponding time series data. The Case-I has been demonstrated in subplots (a)–(c) of Fig. 8. Subplots (d)–(f) of the same figure represent the Case-II. In both case-I and case-II, we have considered two types of initial perturbation, i.e., $d/a = 1.50$ (red line) and $d/a = 1.51$ (blue line).

In subplot (a) of Fig. 8, it is seen that initially, up to $\omega _{pd}t \approx 50$, the particle oscillates sinusoidally with a constant amplitude around a mean value of $z/a \approx 8.9$, i.e., around the background monolayer crystal. However, as time goes on, periodic sinusoidal waveform breaks into a train of pulses, forming envelope structures for both the values of *d*. This is a manifestation of amplitude modulation (AM) of the oscillatory motion of the perturbed particle. If we initially displace all the particles from their equilibrium positions in the vertical direction, the entire monolayer crystal exhibits sinusoidal periodic oscillation around its equilibrium position. In that case, the structural configuration in the *x*–*y* plane of the monolayer crystal does not suffer any change, and the sinusoidal form of the vertical oscillatory motion of the particle does not break into a train of pulses. Thus, the finite boundary of the perturbed region, where the initially perturbed particles and the unperturbed particles of background monolayer crystal interacts, is responsible for such amplitude modulation. For both the values of initial perturbation, i.e., $d/a = 1.50$ (red solid line) and $d/a = 1.51$ (blue dashed line), Fourier spectra of *z* show two side-band frequencies instead of showing a peak at a particular frequency (associated with the external confinement potential). It is also seen that the difference between these two side-band frequencies is $d\omega \approx 0.1\omega _{pd}$ and this is the same for both the cases of initial perturbation. The Fourier spectrum of $z_{max}$ shows a distinct peak at a particular value of $\omega \approx 0.1\omega _{pd}$ for both the values of initial perturbation *d*, as illustrated in subplot (c) of Fig. 8. This corresponds to the frequency of the train of pulses (beat). Thus, the beat frequency originated due to the amplitude modulation of the initial perturbation is the same as the difference between two side-band frequencies of the oscillatory motion of the particles. Therefore, the amplitude modulation of the initial perturbation occurs due to the parametric decay instability, where the frequencies satisfy the three-modes resonance condition, i.e., $\omega _3 = \omega _2 \pm \omega _1$.

It becomes more interesting when we analyze the results at the later stage of evolution (Case II) shown in subplots (d)–(f) of Fig. 8. For the initial perturbation $d/a = 1.50$, the regular train of pulses originated by the amplitude modulation via parametric decay process retains their forms even at the later times of evolution, as can be seen from subplot (d) of Fig. 8. The Fourier spectra of *z* and $z_{max}$ shown in subplots (e)–(f) by solid red lines also confirm our observation. However, a drastic change in the dynamics has been observed in the later stage of evolution due to a slight increase in initial perturbation ($d/a = 1.51$). It is seen from the subplot (d) of Fig. 8 (blue dashed line) that the regular pulse-shaped wave packets, which were formed at initial times due to amplitude modulation, start to deform as time evolves. The amplitude of these pulses also starts to decrease drastically. The Fourier spectra shown in subplots (e) and (f) also reveal this drastic change due to the slight increase of initial perturbation. Two side-band frequencies, which are the signature of amplitude modulation, are still present in the Fourier spectra of *z* for $d/a = 1.50$ (solid red line). On the contrary, for $d/a = 1.51$ (blue dashed line), Fourier spectra of *z* now broaden instead of showing peaks at side-band frequencies. It is seen that at the later stage of time evolution, instead of having a single peak at a particular frequency, which corresponds to the beat frequency, multiple peaks with low amplitudes appear in the Fourier spectra of $z_{max}$ as we increase the initial perturbation from $d/a = 1.50$ to $d/a = 1.51$. This has been depicted in the subplot (f) of Fig. 8. Thus, the Fourier spectra of *z* and $z_{max}$ indicate that multiple irregular beat waves (train of pulses) generate at later times for $d/a = 1.51$.

All the simulation observations presented in this paper can be understood qualitatively from the analysis shown in Fig. 8. In the previous subsection, we have shown that for all the cases of initial perturbation, up to a certain time of evolution, the imparted energy is transmitted from the perturbed central region to the entire crystal through the coherent transverse wavefronts. However, at a later time, only above a certain threshold value of initial displacement ($d_{th}$), the coherent wavefronts are no longer generated. Instead, the initially imparted energy is randomized, resulting melting of the entire crystal. This is the consequence of amplitude modulation of the initial perturbations through parametric decay, as demonstrated in Fig. 8. Here, we have shown that at the initial stage of the evolution, for both the values of initial displacement, the amplitude of the oscillatory motion of perturbed particles gets modulated, forming beat waves with a particular frequency. This is the origin of coherent transverse wavefronts shown in Fig. 2. However, at the later stage of evolution, for a particular value of initial perturbation ($d/a = 1.51$), the coherency of the beat wave breaks into low amplitude multiple pulses with different frequencies. This causes the randomization of initially imparted energy and, essentially, the melting of the entire crystal.

The time evolution of mean kinetic energy associated with the in-plane (${{\hat{x}}}-{{\hat{y}}}$) and out-of-plane (${{\hat{z}}}$) velocity components has been shown in Fig. 9. For both the values of *d*, envelope structures appear in the profile of $K_z$, as shown by the red dashed lines in subplots (a) and (b) of Fig. 9. This is also the consequence of amplitude modulation of vertical oscillations of perturbed particles. It is interesting to observe that for $d/a = 1.50$ [subplot (a)], there is no significant exchange between the in-plane kinetic energy ($K_{xy}$) and out-of-plane kinetic energy ($K_z$). Thus, the mean value of $K_z$ does not change. Only the amplitude of envelopes decreases with time. This is because the vertical oscillation energy of the perturbed central region is transmitted via transverse surface waveforms throughout the crystalline plane without increasing $v_x$ and $v_y$. However, for $d/a = 1.51$, after a certain time, $K_{xy}$ starts to increase drastically at the cost of $K_{z}$, as shown in the subplot (b) of Fig. 9. These observations are consistent with the analysis reported in Fig. 8.

To further characterize the melting dynamics initiated at the 2D crystalline plane, we have considered three cases of initial perturbation: (a) $d/a = 1.51$, (b) $d/a = 1.63$, and $d/a = 1.72$. The in-plane kinetic energy ($K_{xy}$) in the $x-t$ plane, averaged over a narrow strip along ${{\hat{y}}}$ around the middle of the monolayer, has been shown for these three cases in Fig. 10(a)–(c), respectively. It is seen that the distribution of $K_{xy}$ in $x-t$ plane with higher values gets wider as we increase the strength of the initial perturbation. Thus, the velocity of the melting front, which propagates radially outward from the perturbed central region, increases with an increase in initial perturbation strength. In Figs. 8 and 9, it has been revealed that it takes a certain time to initiate melting where a drastic increase of $K_{xy}$ is observed. The delay in initiating the melting is related to the time it takes a parametric decay instability to excite a significant number of unstable modes. This phenomenon has also been captured in Fig. 10. Furthermore, it is also seen that the threshold time to initiate the melting decreases as we increase the strength of the initial perturbation, i.e., with the increase of nonlinearity in the initial perturbation. We have also done Langevin dynamics simulations to include the frictional drag force which is typically present in laboratory dusty plasmas. These results are shown in the Supplementary material.

## Summary

In this work, we have investigated the response of a two-dimensional (2D) crystalline medium under external perturbations. In particular, we have carried out three-dimensional MD simulations to explore the melting process of a 2D ordered structure induced by an initially imposed disturbance. A system of charged particles interacting via Yukawa pair interaction has been considered as a test bed medium. In addition to their pair interaction, particles are also subjected to an effective external potential confining them along the vertical (${{\hat{z}}}$) direction. Under the chosen values of system parameters, it has been shown that particles levitate in a single 2D layer in the *x*–*y* plane, arranging themselves in a crystalline configuration. We imposed a disturbance in this stable crystalline layer by displacing particles initially located within a small circular region around the center of the crystalline plane along the ${{\hat{z}}}$ direction. Since the vertical confining potential profile has a parabolic form, the displaced particles exhibit oscillatory motion in the ${{\hat{z}}}$ direction. In our simulations, we have identified that below a certain value of initial displacement, the externally imposed energy transforms into a train of circular wavefronts propagating radially outward in the *x*–*y* plane from the region of initial perturbation. These circular electrostatic waves are transverse in nature, where particles collectively oscillate along the vertical direction. In these cases, it has been shown that the 2D layer retains its crystalline phase with a slight increase of kinetic energy associated with the ${{\hat{x}}}$ and ${{\hat{y}}}$ components of particles’ velocity. However, above a critical value of initial perturbation, it has been shown that the crystalline order of the 2D layer breaks, and a first-order transition from solid to liquid phase occurs. The critical point of phase transition is shown to be depended upon the strength of the initial perturbation. In our study, we have demonstrated that the nonlinearity in amplitude modulation of initial perturbation via parametric decay instability is responsible for the first-order phase transition. Our findings can be the basis of a deeper understanding of stability and phase dynamics of a wider set of two-dimensional strongly coupled systems, e.g., dusty plasma and colloidal medium.

## Supplementary Information

Supplementary Information 1.

Supplementary Information 1.

Supplementary Information 3.
