# Supplementary material for: Parametric decay induced first-order phase transition in two-dimensional Yukawa crystals
Source: Sci Rep. 2022 Nov 28;12:20430. doi: 10.1038/s41598-022-24988-8 (PMC9705321; doi:10.1038/s41598-022-24988-8)
Supplement: Supplementary file 3 — Supplementary Information 3. [file 41598_2022_24988_MOESM3_ESM.pdf]

Supplementary material

The effect of neutral damping, typically present in dusty plasma experiments, on the phase transition phenomena observed in our study has been explored. For this purpose, we have carried out Langevin molecular dynamics simulations using LAMMPS [1]. In these simulations, we have included both the frictional drag force and random kicks to the dust grains by neutral gas atoms. Thus, the net force acting on any ith particle inside the simulation box is given by,

$$m_d \ddot{\mathbf{r}}_i = -Q \sum_{j=1}^{N} \nabla U(\mathbf{r}_i, \mathbf{r}_j) + Q\mathbf{E}_{ext} + m_d \mathbf{g} - \nu m_d \dot{\mathbf{r}}_i + \zeta_i(t), \tag{1}$$

where ν represents the neutral damping frequency and $\zeta_i(t)$ is the random force acting on the ith particle. Here, m_d and Q represent the mass and charge of the particle. The first, second, and third term in the right side of Eq. 1 represent the force due to the Yukawa pair potential, $U(\mathbf{r}_i, \mathbf{r}_j)$, force associated with the the externally applied electric field, $\mathbf{E}_{ext}$, and force due to gravity, respectively.

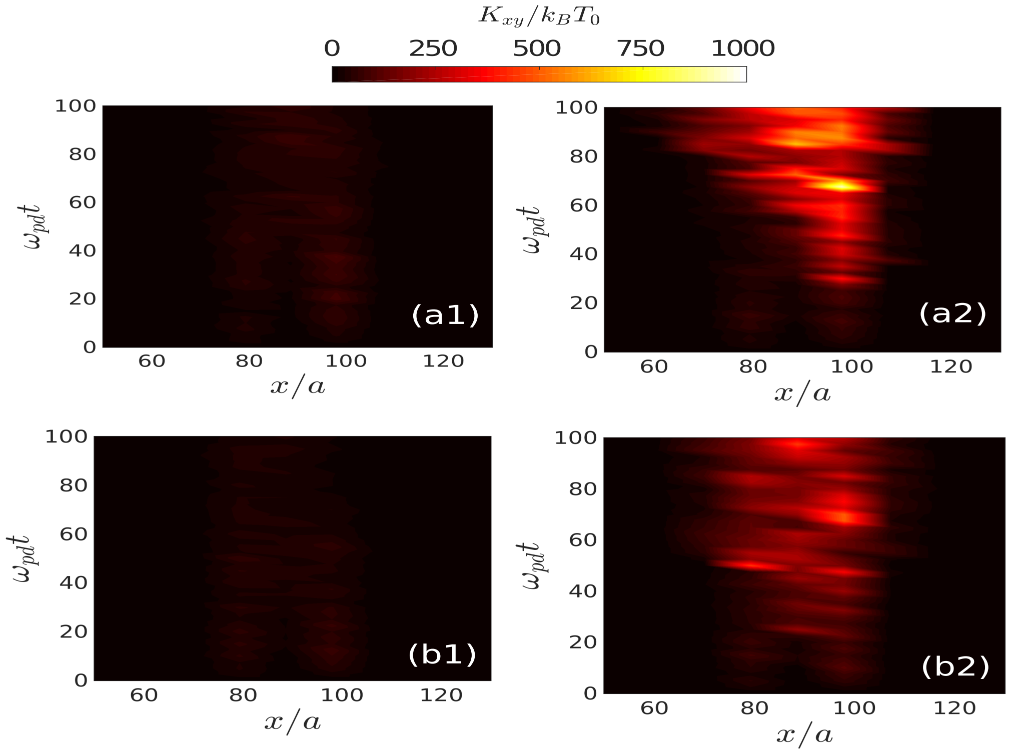

FIG. 1: The distributions of in-plane kinetic energy K_{xy} in $x-t$ plane have been shown for different values of damping coefficient ν and initial perturbation d. The K_{xy} is obtained after averaging over a narrow strip along $\hat{y}$ around the middle of the $x-y$ plane. In subplots (a1) and (a2), the value of ν is kept fixed at $\nu = 0.005\omega_{pd}$ with $d/a = 1.7$ and 1.85, respectively. For subplots (b1) and (b2), damping coefficient ν is kept fixed at $\nu = 0.012\omega_{pd}$ with $d/a = 1.85$ and 2.0, respectively.

The in-plane kinetic energy ($K_{xy} =< \frac{1}{2} m_d (v_x^2 + v_y^2) >$) in the $x-t$ plane, averaged over a narrow strip along $\hat{y}$ around the middle of the monolayer, has been shown in Fig. 1 for two different values of damping coefficient (ν) with various initial perturbations. In one case, we have chosen two different values of $d/a = 1.7$ & $d/a = 1.85$ with a fixed value of $\nu = 0.005\omega_{pd}$ and have been shown in subplots (a1) and (a2), respectively. In another case, we have considered a fixed value of $\nu = 0.012\omega_{pd}$ and two different values of initial perturbation $d/a = 1.85$ and 2.0, as have been shown in subplots (b1) and (b2) of Fig. 1, respectively. It is seen from Fig. 1 (subplots (a2) and (b2)) that in both the

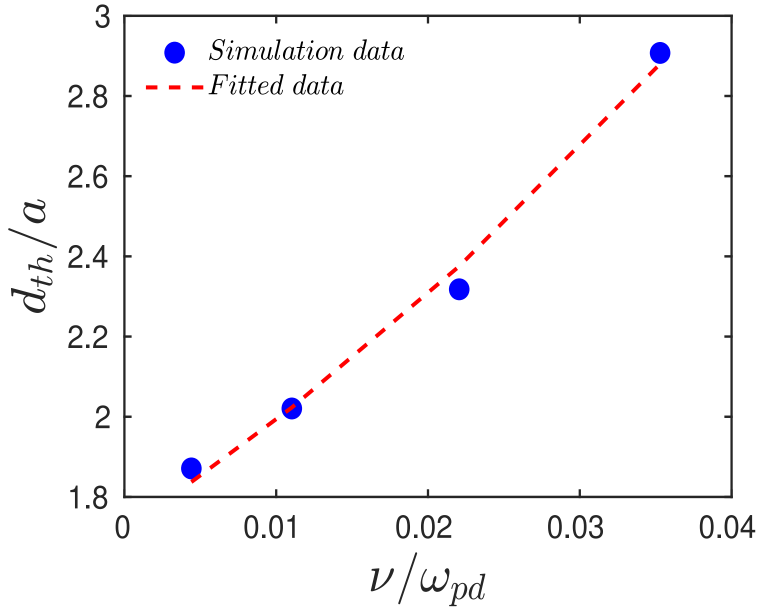

FIG. 2: Variation of the threshold value of initial displacement (d_{th}) for melting with neutral damping frequency ν.

values of ν there is a drastic increase in in-plane kinetic energy (K_{xy}) beyond a threshold value of initial displacement (d_{th}). This demonstrates melting of the crystalline plane.

The variation of the threshold value of initial perturbation (d_{th}) with the neutral damping frequency ν has been shown in Fig. 2. As expected, it is seen that the threshold value of initial perturbation to induce melting increases with the increase of ν. Furthermore, we have also observed in our simulation that for the chosen values of system parameters, there is an upper limit of ν ($\nu \approx 0.05\omega_{pd}$) beyond which the crystal never melts. This may be because beyond this threshold value of ν, the amplitude modulation is insufficient to excite a significant number of unstable modes via parametric decay instability. However, this upper limit of ν will change with the radius of initially perturbed region.

[1] S. Plimpton, Journal of computational physics **117**, 1 (1995).
